# Supplementary material for: Optimizing Time Management for Drip-and-Ship Stroke Patients Qualifying for Endovascular Therapy—A Single-Network Study
Source: Healthcare (Basel). 2022 Aug 12;10(8):1519. doi: 10.3390/healthcare10081519 (PMC9407868; doi:10.3390/healthcare10081519)
Supplement: Supplementary file 1 [file healthcare-10-01519-s001.zip › healthcare-1849192-supplementary.pdf]

Optimizing time management for drip-and-ship stroke patients qualifying for endovascular therapy - a single network experience.

Supplemental Figure S1. Flow chart of included patients. EVT: endovascular therapy, SOS-NET: local stroke network

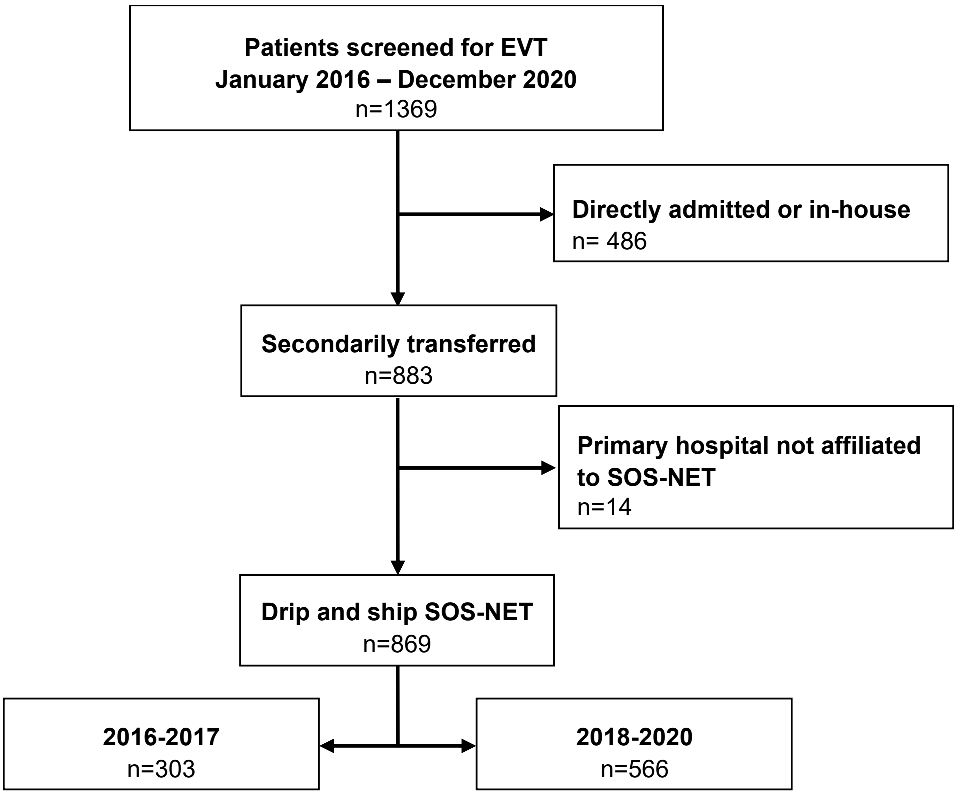

Optimizing time management for drip-and-ship stroke patients qualifying for endovascular therapy - a single network experience.

Supplemental Table S1a. Multiple linear regression for onset to EVT time.

Factors analyzed: admission period, age, sex, hospital, admission time, vascular circulation, intravenous thrombolysis, transfer mode and distance, anesthesia, NIHSS and ASPECTS at comprehensive stroke center admission.

Model		Unstandardized Coefficients		Standardized Coefficients			95,0% Confidence Interval for B		Correlations			Collinearity Statistics	
		B	Std. Error	Beta	t	Sig.	Lower Bound	Upper Bound	Zero-order	Partial	Part	Tolerance	VIF
5	(Constant)	280,713	8,576		32,731	0,000	263,846	297,581					
	admission time = on-call	41,136	7,201	0,282	5,712	0,000	26,973	55,298	0,276	0,291	0,277	0,966	1,035
	distance = 11-30 km	-32,619	7,523	-0,211	-4,336	0,000	-47,413	-17,824	-0,203	-0,225	-0,210	0,989	1,011
	period = 2018-2020	-29,991	7,278	-0,203	-4,121	0,000	-44,305	-15,677	-0,167	-0,215	-0,200	0,972	1,029
	anesthesia = general	18,030	7,885	0,113	2,286	0,023	2,521	33,539	0,128	0,121	0,111	0,957	1,045
	distance = ≤ 10 km	-55,160	25,579	-0,106	-2,156	0,032	-105,468	-4,852	-0,041	-0,114	-0,105	0,973	1,028

a Dependent Variable: onset-EVT start

5

Supplemental Table S1b. Multiple linear regression for primary hospital phase.

Factors analyzed: admission period and time, age, sex, hospital, vascular circulation, intravenous thrombolysis, and transfer mode.

Model		Unstandardized Coefficients		Standardized Coefficients			95,0% Confidence Interval for B		Correlations			Collinearity Statistics	
		B	Std. Error	Beta	t	Sig.	Lower Bound	Upper Bound	Zero-order	Partial	Part	Tolerance	VIF
4	(Constant)	128,694	6,605		19,485	0,000	115,727	141,661					
	vascular circulation = anterior	-46,068	6,160	-0,261	-7,479	0,000	-58,161	-33,975	-0,255	-0,267	-0,261	0,998	1,002
	IVT= no	18,320	3,873	0,166	4,730	0,000	10,716	25,925	0,147	0,173	0,165	0,986	1,014
	admisson time=on-duty	-15,361	3,874	-0,139	-3,965	0,000	-22,967	-7,755	-0,120	-0,145	-0,138	0,983	1,017
	primary hospital=PSC	-8,723	3,802	-0,080	-2,295	0,022	-16,187	-1,260	-0,069	-0,085	-0,080	0,991	1,009

a. Dependent Variable: Primary Hospital

Optimizing time management for drip-and-ship stroke patients qualifying for endovascular therapy - a single network experience.

Supplemental Table S1c. Multiple linear regression for transfer phase.

Factors analyzed: admission period and time, age, sex, hospital, vascular circulation, intravenous thrombolysis,, transfer mode and distance.

Model		Unstandardized Coefficients		Standardized Coefficients			95,0% Confidence Interval for B		Correlations			Collinearity Statistics	
		B	Std. Error	Beta	t	Sig.	Lower Bound	Upper Bound	Zero-order	Partial	Part	Tolerance	VIF
8	(Constant)	68,949	2,381		28,958	0,000	64,275	73,622					
	distance= >100 km	31,060	2,364	0,507	13,139	0,000	26,420	35,700	0,531	0,415	0,334	0,434	2,306
	distance= 11-30 km	-15,677	2,061	-0,287	-7,605	0,000	-19,724	-11,631	-0,472	-0,255	-0,193	0,452	2,214
	distance= ≤10 km	-41,294	5,020	-0,220	-8,227	0,000	-51,147	-31,441	-0,230	-0,274	-0,209	0,898	1,114
	distance= 71-100 km	18,691	2,762	0,224	6,768	0,000	13,271	24,112	0,169	0,228	0,172	0,591	1,692
	transfer mode= ambulance	6,029	1,767	0,116	3,412	0,001	2,561	9,497	-0,327	0,117	0,087	0,563	1,777
	vascular circulation = posterior	4,899	2,040	0,061	2,401	0,017	0,894	8,904	0,087	0,083	0,061	0,994	1,006
	distance= 51-70 km	5,001	2,399	0,074	2,084	0,037	0,291	9,710	-0,011	0,072	0,053	0,509	1,966
	period= 2018-2020	-2,717	1,381	-0,050	-1,967	0,050	-5,429	-0,006	-0,066	-0,068	-0,050	0,981	1,019

a. Dependent Variable: Transfer

Supplemental Table S2. Transfer times according to distance and mode.

Distance, km (n)	Phase	Ambulance n=518, median (IQR)	Helicopter n=351, median (IQR)	P-value
≤ 10	Transfer phase (min)	35 (26-39)	-	
	Request – Departure PH (min)	22 (16-26)	-	
	Departure PH – Door CSC (min)	13 (8-15)	-	
11-30	Transfer phase (min)	57 (49-63)	71 (64-98)	**0.004**
	Request – Departure PH (min)	27 (21-33)	46 (32-58)	**0.001**
	Departure PH – Door CSC (min)	29 (24-33)	33 (23-38)	0.378
31-50	Transfer phase (min)	67 (63-77)	76 (69-88)	**0.004**
	Request – Departure PH (min)	27 (22-31)	51 (42-61)	**0.000**
	Departure PH – Door CSC (min)	40 (36-46)	22 (19-28)	**0.000**
51-70	Transfer phase (min)	71 (64-84)	72 (60-84)	0.471
	Request – Departure PH (min)	28 (23-36)	41 (32-55)	**0.000**
	Departure PH – Door CSC (min)	44 (38-51)	29 (26-33)	**0.000**
71-100	Transfer phase (min)	92 (85-107)	80 (67-94)	**0.001**
	Request – Departure PH (min)	28 (18-47)	47 (35-60)	**0.000**
	Departure PH – Door CSC (min)	65 (61-71)	32 (27-39)	**0.000**
> 100	Transfer phase (min)	114 (103-126)	94 (81-106)	**0.000**
	Request – Departure PH (min)	37 (31-55)	51 (40-63)	**0.000**
	Departure PH – Door CSC (min)	79 (68-91)	39 (35-46)	**0.000**

PH: primary hospital, CSC: comprehensive stroke center
